# Associations between Psychopathology in Mothers, Fathers and Their Children: A Structural Modeling Approach

**DOI:** 10.1007/s10826-018-1024-5

**Published:** 2018-03-01

**Authors:** D. Weijers, F. J. A. van Steensel, S. M. Bögels

**Affiliations:** 1 Kohnstamm Institute, University of Amsterdam, Amsterdam, The Netherlands; 2 Research Institute Child Development and Education, Research Priority Area Yield, University of Amsterdam, Amsterdam, The Netherlands; 3 UvA minds, Academic Treatment Center for Children and Parents, Amsterdam, The Netherlands

**Keywords:** Child psychopathology, Parental psychopathology, Internalizing behavior problems, Externalizing behavior problems, Parenting stress

## Abstract

This study investigated associations between parental and child psychopathology with parenting stress as a possible mediator, in order to get more insight in mothers’ and fathers’ roles in the development of psychopathology in children. Parents of 272 clinically referred (aged 6–20, 66% boys) reported about their own and their child’s behavioral problems, and about parenting stress. Data were analyzed using Structural Equation Modeling. Outcomes of path models demonstrated that mothers’ higher internalizing and externalizing problems were associated with respectively children’s higher internalizing and externalizing problems. Fathers’ higher externalizing problems were associated with both children’s higher internalizing and externalizing problems, but fathers’ internalizing problems were only associated with children’s lower externalizing problems. Parenting stress fully mediated the relation between mothers’ and children’s externalizing problems, and partly mediated the relation between mothers’ and children’s internalizing problems. For fathers, parenting stress partly mediated the relation between fathers’ internalizing problems and children’s externalizing problems. Findings indicate that for mothers, the association between parental and child psychopathology is specific, whereas for fathers it is non-specific. Furthermore, results suggest that reducing parenting stress may decrease child problem behavior. Longitudinal studies are needed in order to gain more insight in the direction and underlying mechanisms of the relation between parental and child psychopathology, including parental stress.

Worldwide, about 13% of the children suffer from mental disorders such as anxiety disorders, depressive disorder, and ADHD (see meta-analysis of Polanczyk et al. 2015). How these mental disorders precisely develop has yet to be discovered, however, a biopsychosocial model (Engel 1980) consisting of child-, parent-, family- and other environmental factors is commonly used. That is, child characteristics (gender, age, IQ), parental characteristics (psychopathology) and parenting rearing practices, family structure, -organisation and system dynamics, having social support and friends, etc., all interact and play a role in child (mal)adaptation (e.g., mental disorder). Similar, Cummings et al. (2000) describe psychopathology as a dynamic interplay of a constantly changing individual in an ever changing environment. Considering child psychopathology, the role of the parent(s) has gained much attention and several ways have been suggested how parents may play a role. First, parents pass their genes to their offspring which makes their child more or less susceptible to a particular disorder. For example, autism developmental disorders seem to have a high genetic component with heritability indices up to 0.92 (e.g., see review of Miles 2011). Second, parents use parenting strategies that may contribute to the development (or maintenance) of childhood mental disorders. For example, parental control has been found to be associated with childhood anxiety (meta-analysis of McLeod et al. 2007; Van Der Bruggen et al. 2008) and corporal punishment has been associated with several child outcomes (e.g., increased child delinquent and antisocial behaviour, decreased mental health; see meta-analysis of Gershoff 2002). Also, children may learn certain behaviours from parents (e.g., via conditioning or operant learning procedures like punishment or reinforcement, or via imitation or listening). Third, parents own characteristics and coping, personality and/or psychopathology may play a role. For example, parental stress has been found to predict child behaviour problems (e.g., Ashfort et al., 2008). Thus, parents may play a role in the development of their child’s psychopathology in multiple ways, and presumably these different ways interact and may exacerbate each other. It may therefore be hardly surprising that research has demonstrated consistently that parental psychopathology and child psychopathology are associated (e.g. Connell and Goodman 2002; Ha et al. 2008; Hodge et al. 2010; Hicks et al. 2004; Van Meurs et al. 2009).

Associations between parent and child psychopathology can be specific (i.e. specific parental disorders are associated with specific disorders in their children), or non-specific (i.e. having a parent with a mental disorder is associated with a higher risk for children to develop any mental disorder), and both have gained some empirical support. For example, with regard to specific associations, it has been found that anxious parents are more likely to have anxious children and vice versa (e.g., Beidel and Turner 1997; Last et al. 1991), and anxious/depressed behavior, somatic problems and rule breaking behavior of the child was best predicted by the same problem behavior of the parent (Van Meurs et al. 2009). However, regarding non-specificity, it has been found that children who have depressed mothers are at elevated risk of developing not only depression themselves, but also conduct behavior problems (e.g., Beck 1999; Goodman and Gotlib 1999), and children whose parents have externalizing disorders (conduct disorder or drug/alcohol dependence) are at increased risk for developing both externalizing disorders and internalizing problems (e.g., Bierut et al. 1998; Clarck et al. 1997; Hicks et al. 2004; Luthar et al. 1993). More knowledge about whether associations are specific or not may lead to better insights on how to conceptualize psychopathology and how to develop prevention or treatment programs. For example, if the associations are non-specific, this may lead to a more dimensional view of psychopathology (e.g., see Caspi et al. 2014, who consider a p-factor as a general psychopathology factor for psychiatric disorders) and more general or symptom-broader (at least not disorder-specific) prevention- or treatment programs, than when associations are found to be specific.

There are (at least) two reasons why the associations between parents and their children’s psychopathology may be different for fathers and mothers. First, females have been found to differ from males in the prevalence and symptom presentation of psychiatric disorders (e.g., Alonso et al. 2004; WHO 2002), and second, fathers and mothers may have different roles in child development and child psychopathology. For example, it has been proposed that fathers have the role to challenge the child and prepare it for the outside world, while mothers’ role is to nurture the child (Bögels and Phares 2008; Bögels and Perotti 2011). Partial support for the different roles of fathers and mothers comes from a study in which fathers’ fulfilment of his evolutionary role to challenge the child was linked to less social anxiety in young children (Majdandžić et al. 2014). Thus, theoretically, the parent-child associations may be different for fathers and mothers. However, previous research was mostly focused on mothers, and (the role of) fathers tended to be neglected for a long time (see review of Phares and Compas 1992; and review of Cassano et al. 2006). From studies that have included fathers (alongside mothers), it has become clear that fathers’ psychopathology is (also) associated with child psychopathology: (1) most paternal psychiatric disorders (such as depression and substance use) are associated with an increased risk for the development of emotional and behavioural problems in their children, independent of maternal psychiatric disorders (see review of Ramchandani and Psychogiou 2009), and (2) results of a meta-analysis demonstrated that externalizing problems in fathers and mothers were comparably associated with externalizing problems in their children, however, while internalizing problems of both mothers and fathers were associated with internalizing problems in their children, the association appeared to be stronger for mothers (Connell and Goodman 2002).

A factor that is frequently linked to child psychopathology, is parenting stress. Although different studies use various definitions of parenting stress, most include the parent’s perception of their capacity to cope with the demands of parenthood (Abidin 1992). Research has found associations between parenting stress and child psychopathology, as well as between parenting stress and parental psychopathology (Anastopoulos et al. 1992; Ashford et al. 2008; Costa et al. 2006; Crnic and Greenberg 1990; Morgan et al. 2002; Webster-Stratton and Hammond 1988). It has been hypothesized that parenting stress leads to poor parenting behaviors (e.g., more authoritarian, harsh and negative parenting) and that these parenting practices in turn causes child maladaptation (e.g., see review of Deater‐Deckard 1998). In addition, parents’ psychopathology and personality, among other variables, are hypothesized to play a role in parenting stress (e.g., see Abidin 1990). Further, parents with psychopathology may be more vulnerable to parenting stress (as they have less coping resources), which lead to more negative parenting, and in turn, parental stress may interfere with their abilities to inhibit negative parenting behaviors (e.g., avoidance or withdrawal when they are high on the internalizing spectrum, and/or aggression when they are high on the externalizing spectrum) (e.g., Bögels et al. 2010). Viewed this way, parenting stress may be an important mediator for the association between parent- and child psychopathology. In addition, if parenting stress is indeed found to mediate the association between parent- and child psychopathology, it may be a relatively easy and important target for treatment (e.g., mindful parenting is found to be an effective intervention to manage parenting stress; Bögels et al. 2014).

The present study aims at testing path models in order to examine the strength of the associations between parental psychopathology and child psychopathology, for both mothers and fathers. In addition, parenting stress is examined as a potential mediator (see Fig. 1). In line with Connell and Goodman’s meta-analysis (2002) we investigated the broadband syndromes, internalizing and externalizing behaviour problems (as there appears to be a high rate of comorbidity within the broadband syndromes internalizing and externalizing problems). Consistent with the Child Behavior CheckList manual (CBCL; Achenbach and Rescorla 2001), internalizing problems were viewed as behaviours and emotions directed inwards which includes anxiety, somatic and depressive symptoms, and externalizing problems were conceptualized as behaviours and emotions directed outwards which includes aggressive, oppositional and rule-breaking behaviours. Based on previous research (see above), we expected internalizing problems in both mothers and fathers to be associated with internalizing problems in their children, and externalizing problems in both mothers and fathers to be associated with externalizing problems in their children. No other expectations were made as previous research reported inconsistent results and/or is lacking.

**Fig. 1:**
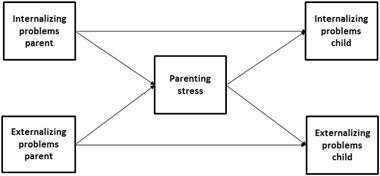
Conceptual model of the mediating role of parenting stress for transmission of psychopathology from parent to child (based on Abidin 1990, and Deater‐Deckard 1998)

## Method

### Participants

The sample of the present study consists of children and their families from an urban area, who were referred to UvA minds, a community mental health care center in the Netherlands, and an academic treatment center for parents and children. The center offers outpatient mental health care to children who have behavioural or emotional problems such as ADHD, anxiety disorders, posttraumatic stress disorder and autism spectrum disorders. All children function on a normal cognitive level (i.e., IQ > 70). Before the family’s first appointment at the treatment center, parents of all children are asked by email to fill in several online questionnaires at home. If they do not complete the online questionnaire at home, they are asked to fill in these forms at the treatment center, before or immediately after their first appointment. Parents are informed about the academic purpose of the treatment center, in which anonymity is guaranteed and have the possibility to resign from participation in the study.

### Procedure

Data were gathered from July 2010 till the end of June 2012 during which 414 mothers and 306 fathers completed the questionnaires. For the current study, data were used when (1) both biological parents participated in the research, and (2) the child was at least 6 years (i.e., for children under 6 a different (preschool) version of the questionnaire on child behavior was used). There were no exclusion criteria. That is, all parents of the children who were referred were asked to complete the questionnaires, irrespective of the reason for referral or diagnosis. Two families did not give their consent to use the completed questionnaires for research aims and their data were therefore removed. The final sample consisted of 272 children (*n* = 180, 66% boys, mean age = 10.35, *SD* = 2.80, range = 6–20 years), 272 mothers (mean age = 42.97 years, *SD* = 5.28, range 24- 57 years) and 272 fathers (mean age = 45.65 years, *SD* = 5.98, range 28–64 years). Of the total sample, 82% of the parents were living together. Ethnicity was based on the mothers’ and fathers’ country of origin. Concerning the educational level of the parents, 66% of the mothers and fathers had a bachelor or master degree. The ethnic composition of the sample of children was 71% Dutch, 17% mixed (Dutch and another country), 12% other (Morocco, Surinam, Turkey, and other western and non-western countries), and 1% unknown.

### Measures

#### Child psychopathology

Children’s internalizing and externalizing behavior problems were measured with the Child Behavior Checklist (CBCL; Achenbach and Rescorla 2001). The questionnaire contains 113 items which are rated on a 3-point Likert scale (0 = ‘not true’, 1 = ‘sometimes true’ or ‘somewhat true’, 2 = ‘often true’ or ‘very true’). The narrowband scales anxious/depressed behavior, withdrawn behavior and somatic complaints were used to form the latent construct internalizing problems. The narrowband scales aggressive behavior and rule breaking behavior were used to form the latent construct externalizing problems. Good reliability and validity of the American version of the CBCL was confirmed for the Dutch translation (De Groot et al. 1994). CBCL *T*-scores were used in order to make the scores between children of different ages and gender comparable. Both mothers and fathers responded on the questionnaire, therefore their mean scores were used. Cronbach’s alphas were calculated for the subscales for both mothers and fathers reports. The alpha ranged between .72 (somatic problems) and .81 (anxious/depressed behavior) for mother report. For father report, the alpha ranged between .69 (rule breaking behavior) and .90 (aggressive behavior).

#### Parent psychopathology

Mothers and fathers reported about their own internalizing and externalizing problems by completing the Adult Self Report (ASR, Achenbach and Rescorla 2003; Ferdinand et al. 1995). The ASR is a 123 item questionnaire, based on the CBCL. The narrowband scales anxious-depressed behavior, withdrawn behavior and somatic complaints were included in the latent construct internalizing problems, while the narrowband scales aggressive behavior, rule breaking behavior and intrusive behavior were included in the latent construct externalizing problems. ASR *T*-scores were calculated. Cronbach’s alpha for the ASR subscales ranged between .72 (withdrawn behavior) and .90 (anxious/depressed behavior) for mother report. The alpha ranged between .62 (rule breaking behavior) and .90 (anxious/depressed behavior) for father report.

#### Parenting stress

Parenting stress was measured with the competence scale of the Nijmegen Parenting Stress Index (De Brock et al. 1992) assessing the degree to which the parent feels capable in dealing with the child. Mothers and fathers filled in the questionnaire about their own experiences of parenting stress. The competence subscale consists of 15 items, such as “I have many more problems raising children than I expected”. Parents rated whether they agreed on the questions on a 6-point Likert-scale, ranging from (1) ‘completely disagree’ to (6) ‘completely agree’. A higher score indicates higher feelings of stress experienced by the parent concerning the parents’ perceived capabilities in parenting the child. For mothers and fathers, scores above respectively 31and 33 can be interpreted as above average. Cronbach’s alphas for maternal and paternal parenting stress in this study were respectively .87 and .90.

### Data Analyses

**Fig. 2:**
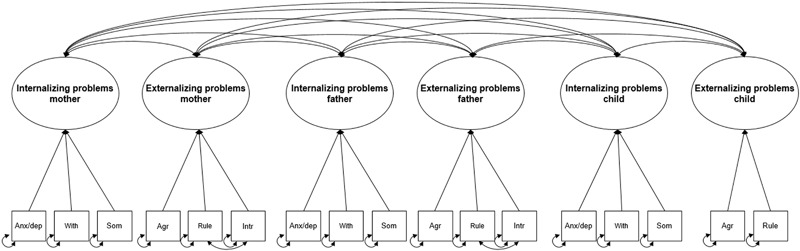
Factor model of internalizing and externalizing problems for mothers, fathers and their children. Latent variables are represented in a circle, observed variables are represented in a square. *Anx/dep* anxiety/depression, *With* withdrawn behavior, *Som* somatic complaints, *Agr* aggressive behavior, *Rule* rule-breaking behavior, *Int* intrusive behavior

In order to analyze the proportion of children and parents that fell in the subclinical (scores between *T*-score ≥ 60 and *T*-score ≤ 63) and clinical range (scores above *T*-score > 63), the scales internalizing and externalizing behavior problems were constructed by calculating the sum score of their narrowband scales. Structural Equation Modeling (SEM) was used for the evaluation of the research questions. SEM was used because it is an appropriate statistical method for mediation analyses and factor analyses with latent variables. Observed covariance matrices were used as input for the analyses. The maximum likelihood estimation method was used to obtain estimates of factor loadings, covariances and residual variances. Several fit indices were used to evaluate the fit of the factor models (see Fig. 2 for the six-factor model) to the data. The Chi-square (χ²) test is a measure of exact fit. A significant χ²-value at an alpha-level of 0.05 indicates that the model does not fit the data. The χ² however is sensitive to sample size and is not very accurate (Browne and Cudeck 1992), and therefore the Root Mean Square Error of Approximation (RMSEA) and the comparative fit index (CFI) were also considered. The RMSEA is a measure of approximate fit. RMSEA values higher than 0.10 indicate bad fit, values lower than 0.08 indicate satisfactory fit, and values lower than 0.05 indicate close fit (Browne and Cudeck 1992). The CFI ranges from 0 to 1, where 1 indicates best fit (Hu and Bentler, 1999). Likelihood based confidence intervals were used to test the significance of the direct and indirect effects. Coefficients of the direct and indirect effects are standardized, and thus values of 0.1, 0.3, and 0.5 can be interpreted as respectively, ‘small’, ‘medium’, and ‘large’ effects (Cohen 1992). The analyses were conducted with the computer program OpenMx (Boker et al. 2011) and based on correlation residuals, i.e. differences between the observed and predicted covariances, exceeding .10, covariances were added to the models. Multiple models were run. First, to investigate the strength of the association between mother–child and father–child psychopathology, separate models were run for the associations between mother–child and father–child psychopathology. Second, to examine whether one of the parents had stronger associations with child psychopathology, i.e., to test the strength of the mother–child and the father-child association while controlling for the effect of the other partner, a structural regression model was constructed in which the psychopathology of both parents was included in the same model. Third, in order to test whether parenting stress mediated the relation between parental and child psychopathology, parenting stress was added to the structural regression models for both fathers and mothers separately. In the mediation models, direct effects represent the associations between parent psychopathology (maternal and paternal internalizing and externalizing problems) and child psychopathology (the child’s internalizing and externalizing problems), while indirect effects represent the mediating influence of parenting stress (thus, whether or not the association between parent and child psychopathology is—partly or fully—explained by parenting stress). It would have been interesting to see the indirect effect via parenting stress in the model with mothers and fathers together, however, this model did not converge.

## Results

### Severity of Behavior Problems and Parental Stress

The average ratings of children’s internalizing problems fell in the subclinical range, *M* = 60.63 (*SD* = 8.92). The average ratings of children’s externalizing problems fell in the normal range, *M* = 56.28 (*SD* = 9.38). Of the total sample 30% of the children were reported by their parents to have scores falling within the normal range for both internalizing as well as externalizing problems. The percentage of children who’s internalizing or externalizing behavior problems were rated in the subclinical range, using the mean scores of the mothers and fathers, were respectively 17 and 12%. The percentage of children who’s internalizing or externalizing behavior problems were rated in the clinical range, were respectively 42 and 24%.

The mean scores of parents’ internalizing problems (*M*_mothers_ = 49.25, *SD* = 10.47, *M*_fathers_ = 48.23, *SD* = 11.45), and externalizing problems (*M*_mothers_ = 48.06, *SD* = 9.54, *M*_fathers_ = 47.54, *SD* = 9.91) fell in the normal range. The scores did not differ statistically between mothers and fathers. Of the parents, respectively 16 and 11% rated themselves as having internalizing and externalizing problems in the subclinical or clinical range. The mean levels of parenting stress reported by mothers and fathers, were respectively 32.88 (*SD* = 11.57) and 31.77 (*SD* = 11.96), and were not statistically different. The mean scores indicate that on average, both mothers and fathers appeared to experience above average parenting stress. The percentages of mothers and fathers reporting above average parenting stress were respectively 48 and 49%.

### Associations between Parent and Child Psychopathology

The six-factor model is presented in Fig. 2. The model consisted of the factors internalizing problems and externalizing problems of mothers, fathers and children. See Table 1 for the correlation matrices with standard deviations of the observed variables included in the factor and structural regression models. See Table 2 for the correlations between the latent factors internalizing problems and externalizing problems of parents and children.

**Table 1:** Correlations among parental psychopathology, child psychopathology and parental parenting stress

	1.	2.	3.	4.	5.	6.	7.	8.	9.	10.	11.	12.
1. Anx/dep parent	–	.66**	.60**	.64**	.41**	.17**	.20**	.08	.18**	.02	.04	.49**
2. With parent	.55**	–	.43**	.49**	.36**	.09	.15*	.17**	.18**	.07	.10	.45**
3. Som parent	.54**	.26**	–	.55**	.34**	.22**	.21**	.15*	.34**	.17**	.12	.36**
4. Agr parent	.62**	.37**	.45**	–	.48**	.41**	.27**	.16**	.26**	.20**	.16**	.42**
5. Rule parent	.34**	.31**	.31**	.46**	–	.36**	.14*	.07	.13*	.12	.20**	.28**
6. Intr parent	.25**	.12	.30**	.42**	.39**	–	.20**	.11	.11	.19**	.19**	.15*
7. Anx/dep child	.24**	.13*	.19**	.20**	.10	.09	–	.46**	.47**	.29**	.16**	.27**
8. With child	.12*	.14*	.09	.14*	.09	.05	.46**	–	.36**	.17**	.17**	.19**
9. Som child	.20**	.08	.32*	.16**	.10	.09	.47**	.36**	–	.17**	.21**	.19**
10. Agr child	.15*	.01	.11	.20**	.16**	.12*	.29**	.17**	.17**	–	.67**	.29**
11. Rule child	.13*	.05	.13*	.20**	.17**	.06	.16**	.17**	.21**	.67**	–	.28**
12. Stress	.45**	.34**	.27**	.46**	.27**	.10	.25**	.27**	.21**	.35**	.41**	–
*M* (*SD*) Mothers	54.23 (6.65)	53.33 (5.17)	54.35 (5.55)	54.18 (5.30)	53.61 (5.64)	51.67 (3.55)	60.56 (8.10)	61.65 (8.93)	58.60 (7.11)	58.81 (7.87)	56.03 (5.78)	32.87 (11.57)
*M* (*SD*) Fathers	53.97 (6.47)	54.38 (6.59)	53.40 (5.39)	54.43 (6.17)	53.53 (4.60)	51.47 (3.15)						31.77 (11.96)

*Note*: The correlations above the diagonal concern the fathers, the correlations under the diagonal concern the mothers.

*Anx* anxiety/depression, *With* withdrawn behavior, *Som* somatic complaints, *Agr* aggressive behavior, *Rule* rule-breaking behavior, *IntR* intrusive behavior, *Stress* parenting stress. *M* mean, *SD* standard deviation

*p < .05, **p < .001

**Table 2:** Correlations among the latent variables internalizing problems and externalizing problems of parents and children

	1.	2.	3.	4.	5.	6.
1. Internalizing mother	–					
2. Externalizing mother	.73*	–				
3. Internalizing father	.27*	.18*	–			
4. Externalizing father	.21*	.15*	.77*	–		
5. Internalizing child	.33*	.28*	.31*	.38*	–	
6. Externalizing child	.17*	.26*	.07	.25*	.36*	–

*p < .05

**Fig. 3:**
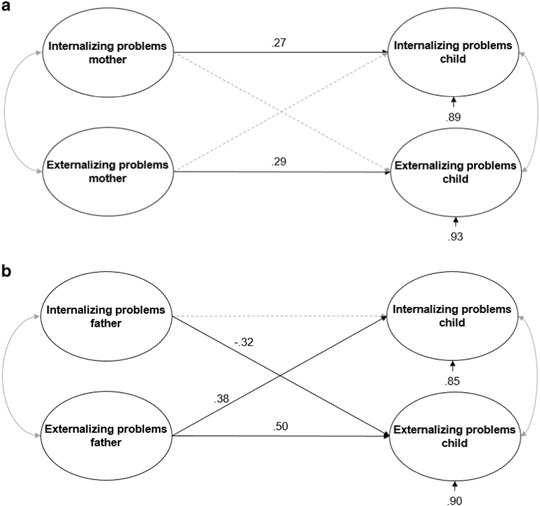
**a** Model for the relation between maternal and child psychopathology. The dotted line represent the non-significant effect. The propotions of unexplained variance are shown under the dependent variables. **b** Model for the relation between paternal and child psychopathology. The dotted line represent the non-significant effect. The propotions of unexplained variance are shown under the dependent variables

Figure 3a, b display the models in which the strength of the association of mothers’ and fathers’ psychopathology with child psychopathology is examined. The model for mothers (χ² (37) = 67.788 =, *p* = .001, RMSEA = .055 (95% CI = [.028, .079]), CFI = .96) showed two small-sized associations (more internalizing problems in mothers was related to more child internalizing problems, *β* = .27, *p* < .05; and more externalizing problems in mothers was related to more child externalizing problems, *β* = .29, *p* < .05). The model explained 11% of the child’s internalizing problems, and 7% of the child’s externalizing problems. For fathers (χ² (37) = 89.896 =, p = .000, RMSEA = .066 (95% CI = [.042, .089]), CFI = .95), one large-sized association (more externalizing problems in fathers was related to more child externalizing problems, *β* = .50, *p* < .05) and two medium-sized relations (more externalizing problems in fathers was related to more child internalizing problems, *β* = .38, *p* < .05; and more internalizing problems in fathers was related to less child externalizing problems, *β = *−.32, *p* < .05) were found. The model explained 15% of the child’s internalizing problems, and 10% of the child’s externalizing problems.

**Fig. 4:**
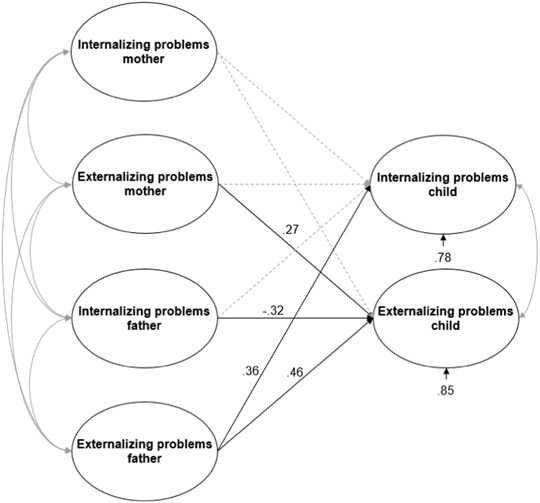
Model for the relation between parental and child psychopathology. The dotted line represent the non-significant effect. The propotions of unexplained variance are shown under the dependent variables

Figure 4 displays the structural regression models in which the strength of the association of mothers’ and fathers’ psychopathology with child psychopathology can be compared to each other. Concerning the RMSEA, there was satisfactory fit of the model to the observed covariance matrix, χ² (102) = 210.947, *p* = .000, RMSEA = .063 (95% CI = [.048, .077]), CFI = .93. Results of the combined parents model were similar to the separate mother-child and farther-child models with the exception that the positive association between mothers’ internalizing problems and children’s internalizing problems no longer reached significance. The small-sized positive association between mothers’ externalizing problems and children’s externalizing problems (*β* = .27, *p* < .05) remained. With respect to fathers, results were similar to the father-child model: two medium-sized positive associations (between fathers’ externalizing problems and children’s externalizing problems, *β* = .46, *p* < .05; and between fathers’ externalizing problems and children’s internalizing problems, *β* = .36, *p* < .05), and a medium-sized negative association (between fathers’ internalizing problems and children’s externalizing problems, *β = *−.32, *p* < .05) were found. The model explained 22% of the child’s internalizing problems, and 15% of the child’s externalizing problems.

### Parenting Stress as Mediator

**Fig. 5:**
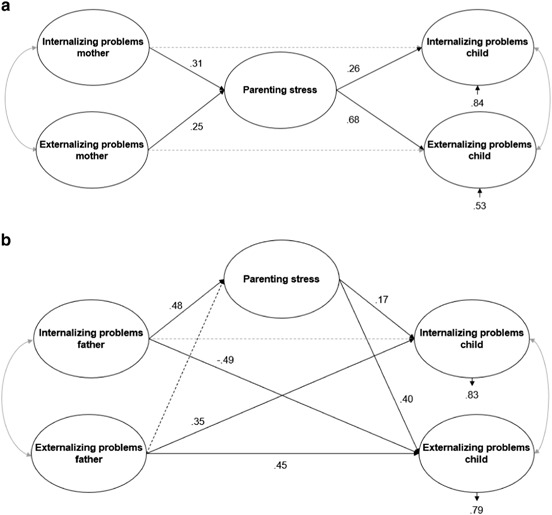
**a** Model for the relation between maternal psychopathology, child psychopathology and parenting stress. The dotted line represent the non-significant effect, suggesting full mediation. The propotions of unexplained variance are shown under the dependent variables. **b** Model for the relation between paternal psychopathology, child psychopathology and parenting stress. The dotted lines represent the non-significant effects. The propotions of unexplained variance are shown under the dependent variables

Figures 5a and 5b show the partial mediation models for the relation between parental and child psychopathology via parenting stress for mothers (χ² (39) = 67.678, *p* = .003, RMSEA = .052 (95% CI = [.025, .076]), CFI = .97) and fathers (χ² (44) = 92.66, *p* = .000, RMSEA = .064 (95% CI = [.042, .085]), CFI = .96) separately. Mothers’ parenting stress was a small but significant mediator for the association between mothers’ internalizing problems and both children’s internalizing (*β* = .08, *p* < .05) and externalizing problems (*β* = .21, *p* < .05), and for the association between mothers’ externalizing problems and both children’s externalizing (*β* = .17, *p* < .05) and internalizing problems (*β* = .07, *p* < .05). The direct effects between mothers' (internalizing and externalizing) problems and children’s (internalizing and externalizing) problems were not significant. The model explained 16% of the child’s internalizing problem behavior, and 47% of the child’s externalizing problem behavior.

In the mediation model for fathers, a medium-sized negative direct effect between fathers’ internalizing problems and children’s externalizing problems (*β* = −.49, *p* < .05) was found, and this relation was found to be mediated by parenting stress. Contrary to the negative direct effect of fathers’ internalizing problems on children’s externalizing problems (i.e., more internalizing problems in fathers was related to less externalizing problems in their children), the indirect effect of paternal internalizing problems via parenting stress was positive, but small (*β* = .20, *p* < .05). Furthermore, there was a medium-sized positive direct effect of fathers’ externalizing problems on children’s externalizing problems (*β* = .45, *p* < .05), and children’s internalizing problems (*β* = .35, *p* < .05) (i.e., more externalizing problems in fathers were related to more externalizing and internalizing problems in their children). The model explained 17% of the child's internalizing problem behavior, and 21% of the child's externalizing problem behavior. A mediation effect of parenting stress for the association between fathers’ externalizing problems and children’s psychopathology was not tested, since fathers’ externalizing problems did not appear to be associated with fathers’ parenting stress.

## Discussion

This study examined the associations between parental psychopathology and child psychopathology with the use of Structural Equation Modeling, and investigated the mediating effect of parenting stress. The results of this study showed that psychopathology in both mothers and fathers is substantially associated with psychopathology in children. However, the pathways of these associations differed for mothers and fathers. That is, specificity was found for mothers (mothers’ internalizing problems were positively related to child internalizing problems and the same applied for externalizing problems), whereas non-specificity was found for fathers (fathers’ externalizing problems were positively related to both child internalizing- and externalizing problems, and fathers’ internalizing problems were negatively related to child externalizing problems). Considering the strength of the associations of mothers and fathers in the combined parent model, it was found that the associations between fathers’ and children’s psychopathology was stronger than between mothers’ and children’s psychopathology. Moreover, the relation between mothers’ and children’s internalizing problems disappeared when fathers’ psychopathology was controlled for. Regarding parenting stress, it was found that maternal parenting stress fully mediated the associations between maternal psychopathology and child psychology, while for fathers, parenting stress was only found to mediate the association partly and direct effects remained. Taken the results of all models together, it appears that mothers’ internalizing problems only function as a risk factor for the development of child (internalizing) psychopathology when fathers' psychopathology is not accounted for, and that mothers’ externalizing problems only play an indirect role via parenting stress. Instead, fathers’ externalizing problems do appear to function as an important risk factor for child (internalizing and externalizing) psychopathology, whereas their internalizing problems appeared to be a protective factor for child externalizing problems.

Concerning child internalizing problems, no associations were found with parental internalizing problems which is in contrast to the finding of a small, but significant, association between both maternal-child and paternal-child internalizing problems (e.g., see meta-analysis of Connell and Goodman 2002), and in contrast to the studies that have demonstrated that anxious parents are more likely to have anxious children and vice versa (see review of Ginsburg and Schlossberg 2002). An explanation of the contrast in our findings relative to previous studies is that we included both maternal and paternal psychopathology in the same model, while previous studies analyzed mother and father data separately. Important to add here is that—in line with previous studies (e.g., meta-analysis of Connell and Goodman 2002)—in the separate mother-child model, a small but significant positive relation was found between maternal and child internalizing problems. However, in the separate father-child model this relation was not significant. What was found to be significant—in both the separate father–child model as well as the combined parent model—was the relation between paternal externalizing problems and child internalizing problems. Thus, more externalizing problems in fathers was related to more child internalizing problems. Note that more externalizing problems in fathers was also associated with more child externalizing problems, suggesting that fathers’ externalizing problems may be a more general risk factor to child (internalizing as well as externalizing) behavioral problems. Alternatively, fathers with externalizing problems may be less involved, and paternal involvement has been found to be associated with child internalizing as well as externalizing behavioral problems (e.g., see review of Barker et al. 2017).

Concerning externalizing problems in children, three significant associations were found. First, in line with previous research (e.g., meta-analysis of Connell and Goodman 2002), a positive association for externalizing problems was found between mothers and their children, however, it was also found that this association was fully mediated by maternal parenting stress. Thus, externalizing problems in mothers may have an indirect effect (via parenting stress) rather than a direct effect on their child’s externalizing behaviors. In support, higher levels of parenting stress have been found to be associated with more dysfunctional parenting, which in turn is related to more child problem behaviors (e.g., see review of Morgan et al. 2002). Second, fathers’ externalizing problems were found to be associated with their child’s externalizing problems, and—in contrast to the results of mothers—these associations were only partly mediated by parenting stress. Explanations that may be offered for the difference in the direct and indirect effect in the mother–child association versus the father–child association are: (a) the direct effect between fathers’ and children’s externalizing problems may suggest a stronger genetic component in the father to child transmission of “externalizing genes” and/or fathers are more a role model when it comes to externalizing behavior such as displaying aggression—of which partial support comes from studies that have found males to exhibit more externalizing problems than females (Alonso et al. 2004; WHO 2002), and/or (b)—as the study used a cross-sectional design and no causal interferences can be made—the fully mediated effect of parenting stress in mothers may be explained by a higher susceptibility for mothers than fathers to child psychopathology and parenting stress. Support for this explanation comes from research involving parents of children with various disorders (e.g., autism spectrum disorder, ADHD, disruptive behavior) that demonstrated higher levels of parenting stress in mothers than in fathers (Baker 1994; Calzada et al. 2004; Moes et al. 1992; Oelofsen and Richardson 2006). Third, fathers internalizing problems were found to have a direct effect on children’s externalizing behaviors, but in the way that paternal internalizing problems may serve as a protective factor for children’s externalizing behaviors. Possibly, fathers with more internalizing problems have children who are more inhibited or less sensation-seeking, and therefore these children may be less susceptible for developing externalizing problems (Kimonis et al. 2006; Williams et al. 2009).

A specific strength of this study is the inclusion of both fathers and mothers, and the possibility to examine both separate mother–child and father–child models (to examine the strength of the association between mother/father and child psychopathology) as well as a combined parent model (to examine the parent-child associations while controlling for the effect of the other partner). Another strength is that this study was based on a large clinical sample which was not self-selected. This makes the results relevant for clinical practice, since the outcomes can be generalized to a large group of children with emotional-behavioral problems that are in need for treatment. However, limitations also need to be considered. First, the cross-sectional design of the current study did not provide the opportunity to consider the direction of the relation between parental and child psychopathology. The focus of this paper was to see whether parental psychopathology and child psychopathology are related and the starting point was the pre-assumption that parent psychopathology (whether or not mediated by parenting stress) influenced child psychopathology. However, likely, bi-directional relations between parent and child psychopathology, between parent psychopathology and parenting stress, and between parenting stress and child psychopathology exists (e.g., Pettit and Arsiwalla 2008; Neece et al. 2012) and longitudinal studies are necessary to investigate the causality between these factors. Second, only parents reported on children’s problem behavior, which could have biased the results, since the presence of psychopathology in parents might bias their rating of their child’s behavior (De Los Reyes and Kazdin 2005). Multiple reports and observations of child and parent psychopathology should be included in order to generate more confidence in the results. Third, it was remarkable that 30% of the children were reported by their parents to have scores falling in the normal range for both internalizing and externalizing disorders on the CBCL, while all children were referred to the mental health care center for diagnostics and/or treatment, and all children were diagnosed with at least one disorder according to the DSM-IV-TR (suggesting at least some impairment and/or emotional-behavioral difficulties). An explanation for this finding may be that not all emotional-behavioral problems are captured by the CBCL internalizing or externalizing scales. For example, the externalizing scale of the CBCL does not include the attention problem subscale - while scores on that subscale have been found to be predictive for ADHD (e.g., Hudziak et al. 2004). Likewise, children diagnosed with post-traumatic stress disorder may not score very high on a general measure that assesses child problem behaviors (Sim et al. 2005). Finally, a limitation of the study is that it was restricted to the association between parental psychopathology and child psychopathology and that only parenting stress was examined for its mediating influence. The relation between parent and child psychopathology however is complex (e.g., Cummings et al. 2000; Engel 1980) and depending on multiple variables including child characteristics. As an example McBride et al. (2002) found that the association between child temperament and parenting stress differed not only by the gender of the parent, but also by the gender match of the parent with the child.

With respect to future research, it is important to replicate the findings of this study with the use of a longitudinal design, in order to make causal inferences about the relation between parental and child psychopathology. In addition, research is needed to further investigate mechanisms of the relation between parental psychopathology, child psychopathology and parenting stress, under which the found reverse relation between fathers' internalizing and the child’s externalizing symptoms. Furthermore, exploring the influence of possible mediating or moderating factors such as the child’s gender, parental cognitions, co-parenting and marital functioning, will contribute to more insight in pathways to (mal)adaptive child outcomes. Such relations between the child and its environment can be expected to be interdependent and bi-directional (Garbarino and Ganzel 2000; Sameroff 2010). For example, problem behavior of a parent may lead to more parenting stress, resulting in less supportive parenting, which may result in more problem behavior in their children. In turn, children’s behavior problems may lead to more parenting stress, probably resulting in less supportive parenting as well as more parental behavior problems. From the current study, no interferences can be made with respect to causality or bi-directionality, however, the findings of the current study do highlight the importance of including both mothers and fathers when studying the association between parental psychopathology, child psychopathology and parenting stress.
